# The Board of Health of the State of Rhode Island

**Published:** 1899-10

**Authors:** 


The Board of Health of the State of Rhode Island has drafted a bill which it is endeavoring to get through the Legislature, forbidding the practice of so-called Christian Science. One of the pleas made for the bill is the fact that, with their pecular views and practices, Christian Scientists are public dangers, especially in times of epidemic disease. This is a truth that ought to be widely published. The danger from the sect is not in the occasional passive manslaughter in which they indulge, but in the actual every-day risk to their fellow-beings. One zealous Christian Scientist can undo the work of many efficient health police, provided he has the liberty to carry out his religious medical vagaries to their logical results. It is certainly in the interest of public health that some restriction should be placed upon them, that they should be compelled to at least respect sanitary regulations, regardless of their peculiar views.

